# The influence of body composition on therapeutic hypothermia: a prospective observational study of patients after cardiac arrest

**DOI:** 10.1186/cc6954

**Published:** 2008-07-10

**Authors:** Joost J Jimmink, Jan M Binnekade, Frederique Paulus, Elisebeth MH Mathus-Vliegen, Marcus J Schultz, Margreeth B Vroom

**Affiliations:** 1 Department of Intensive Care Medicine, Academic Medical Centre, University of Amsterdam, 1100DD Amsterdam, The Netherlands – Meibergdreef 9, 1105 AZ Amsterdam; 2 Department of Gastroenterology, Academic Medical Centre, University of Amsterdam, 1100DD Amsterdam, The Netherlands – Meibergdreef 9, 1105 AZ Amsterdam; 3 Laboratory of Experimental Intensive Care and Anesthesiology (L.E.I.C.A.), Academic Medical Centre, University of Amsterdam, 1100DD Amsterdam, The Netherlands – Meibergdreef 9, 1105 AZ Amsterdam; 4 HERMES Critical Care Group, Amsterdam, The Netherlands

## Abstract

**Introduction:**

Patients after out-of-hospital cardiac arrest (OHCA) benefit from therapeutic hypothermia for 24 hours. The time needed to reach hypothermia (target temperature of 32°C to 34°C) varies widely. In this study, we explore the relation between measures of body composition and the time needed to reach target temperature with hypothermia.

**Method:**

We conducted a prospective observational study in patients treated with hypothermia after OHCA. Data collected included weight and height, body composition by anthropometric measures and by single-frequency body impedance, and waist-to-hip ratio. Analysis of concordance between impedance and anthropometric measures and hazard ratios of achieving target temperature (event) corrected for different body composition measures.

**Results:**

Twenty-seven patients were included. The median (interquartile range) time to reach target temperature after admission to the intensive care unit was 191 (105 to 382) minutes. Intraclass correlation for total body fat (TBF) measures was 0.94 (95% confidence interval [CI] 0.89 to 0.97). Only TBF percentage (anthropometrics by the Durnin's table) appeared to be associated with time to reach target temperature: 0.93 (95% CI 0.87 to 0.99; *P *= 0.03).

**Conclusion:**

The body composition measures from single-frequency impedance and anthropometrics appear to be very concordant. Only TBF percentage (anthropometrics) showed a significant but clinically irrelevant influence on time needed to achieve target temperature with hypothermia. We conclude that there are no indications to adjust current cooling practice toward the body composition of patients.

## Introduction

Patients after out-of-hospital cardiac arrest (OHCA) benefit from therapeutic hypothermia for 12 to 24 hours [1,2]. The speed with which therapeutic hypothermia is started seems important for its effect [3,4]. Considering that there is always a delay in reaching the intensive care unit (ICU), the target temperature should be reached as soon as possible, preferably within 30 minutes [5]. Times needed to achieve target temperature with hypothermia (32°C to 34°C) vary widely, from 0.5 to 8 hours or even longer [6]. We encountered a similar variation in our practice. We hypothesized that the variation of the time needed to achieve the target temperature was caused, at least in part, by patient factors such as weight and fat percentage. Therefore, in the present study, we determined the relation between body composition and the temperature course during therapeutic hypothermia. Both anthropometric and bioelectrical impedance measures were used to estimate body composition.

## Materials and methods

### Patients and setting

From May 2006 until June 2007, 27 consecutive OHCA patients who were eligible for therapeutic hypothermia and who were admitted to the 28 bed ICU of the Academic Medical Center, Amsterdam, The Netherlands, were included. The study protocol was approved by the local institutional review board, and a signed informed consent form was obtained from the next of kin for patients eligible for hypothermia. If patients were capable, they gave informed consent after discharge from the ICU.

### Study design

We conducted a prospective observational study of body composition by bioimpedance and anthropometrics and temperature profiles during hypothermia.

### Inclusion of patients

Comatose survivors after OHCA who were admitted to our hospital and who were at least 18 years old were included. Patients eligible for hypothermia but who could not be weighed by a mattress balance (that is, patients in a prone position or instable hemodynamically) were excluded from the study.

### The therapeutic hypothermia protocol

Patients were subjected to the hypothermia protocol. On arrival at the ICU, the patient was placed on a cooling mattress (Blanket roll 2; Cincinnati Sub-Zero Products, Inc., Cincinnati, OH, USA), and at the same time, ice-cold lactated Ringer solutions (4°C) were administered with a rate of 100 mL/minute and a maximum of 30 mL/kg body weight of the patient. Infusions were stopped if the target temperature (32°C to 34°C) was reached. The induced cooling was maintained by using this cooling mattress. When the patient reached the temperature of 34.5°C, the mattress setting was switched from 'manual' to 'auto mode' with a target setting of '33°C'. Temperature was maintained between 32°C and 34°C for 24 hours. The mattress in 'auto mode' used a rectal temperature probe for automatic adjustment of the extent of cooling applied (Blanket roll 2). We also monitored the continuous nasal temperature with a probe connected to the bedside monitor (Philips IntelliVue; Philips, Eindhoven, The Netherlands). Patients were sedated with midazolam and morphine at starting doses of 5 and 2 mg/hour, respectively. All patients were sedated at a Ramsay score of 6 (no response to glabellar tap or loud noise) during the period of hypothermia. Shivering was to be detected by the ICU nurse or the ICU doctor; in case of shivering, muscle relaxation was achieved with deeper sedation and/or rocuronium. After reaching the target temperature, hypothermia was maintained for the next 24 hours, after which patients were passively warmed to a normal temperature.

### Height and weight measurements

Absolute body weight was measured within the first 12 hours of admission using a mattress balance (Sling-scale 2002; Scale-Tronix, White Plains, NY, USA) with an accuracy of 0.05 kg. Length was measured with a tape measure.

### Impedance measurements

Single-frequency bioimpedance was measured using a Bodystat 1500 (Bodystat Ltd, Douglas, Isle of Man, UK). This impedance measures resistance at a single frequency (50 kHz). Calculations of total body water, fat-free mass, and total body fat (TBF) are done with regression equations derived from a resistance index.

### Anthropometric measurements

The TBF and total body lean weight were calculated from the anthropometric measures (Table 1). All measures were performed in triplicate by the researcher (JJJ).

**Table 1:** Anthropometric measures

Measures	Conditions
Skin fold thickness (caliper 5 g/mm^2^)	
Triceps and biceps	Nondominant side/supine position/between olecranon and acromion
Subscapular	Two centimeters below scapula/patient lying on one side
Supra iliac	Upper anterior iliac spine
Dimensions	
Condylar	Breath (sliding caliper)
Bistyloid	Breath (sliding caliper)
Waist	Circumference of minimum waist (cm)
Hip	Circumference of maximum hip (cm)
TBF calculations, percentage	
Womersley [9]	33.5 (log Σ 4 skin fold thickness) - 31.1
James [10]	Men: 1.1 × weight - (128 × weight^2^/100 × height^2^)
	Women: 1.07 × weight - 148 × weight^2^/100 × height^2^
von Döbeln [11]	15.1 × [(height)^2 ^× (Σ femoral condylar breaths) × (Σ radioulnar breaths)] 0.712
Deurenberg *et al*. [12]	(1.2 × BMI) + (0.23 × age) - (10.8 × gender) - 5.4

BMI, body mass index; TBF, total body fat.

### Data collected

We registered the temperature for periods (a) from the start of active cooling until the target temperature was reached and (b) from the termination of active cooling until the time at which the temperature was 36°C. Other data collected were age, gender, prescribed dosages of rocuronium, length of ICU stay and length of hospital stay, APACHE II (Acute Physiology and Chronic Health Evaluation II) score at admission, ICU mortality, and hospital mortality.

### Statistical analysis

Descriptive statistics were used to characterize the study sample. Intraclass correlation coefficients (ICCs) were used to assess the concordance between measures of body composition by impedance and by anthropometrics. The association between the different body measures (Table 2) and the time needed to achieve the target temperature is assessed by the Cox proportional hazards regression analysis. Statistical uncertainties for differences are expressed by the 95% confidence limits. Data were analyzed with a statistical software package (SPSS 12.0.1 for Windows; SPSS Inc., Chicago, IL, USA).

**Table 2:** Body measures

(n = 27)	Mean (SD)	Range (minimum-maximum)
Weight, kg	83 (18)	69 (43–112)
Height, cm	174 (10)	39 (150–189)
Body mass index	27 (6)	22 (16–38)
Body surface area, cm^2^	1.99 (0.25)	1 (1.35–2.35)
TBF by bioimpedance, percentage	26.8 (11.47)	44 (7–51)
TBF by Durnin (table), percentage	27.8 (9.6)	35.5 (12.2–47.7)
TBF by Womersley [9] formula, percentage	25.8 (8.8)	35.1 (6.2–41.3)
TBF by von Doebeln [11], percentage	25.3 (10.6)	35 (4.8–41.1)
TBF by James [10], percentage	26.8 (8.9)	35 (14–49)
TBF by Deurenberg *et al*. [12], percentage	32.8 (9.8)	38.5 (17.1–55.6)
Waist-to-hip ratio	0.97 (0.092)	0.39 (0.77–1.14)

SD, standard deviation; TBF, total body fat.

## Results

### Patients

Twenty-seven consecutive patients admitted to the ICU between May 2006 and June 2007 participated in the study. Patient characteristics are shown in Table 3. Twenty out of 27 patients received rocuronium, and the mean dose (standard deviation, SD) was 104 (82) mg (range 25 to 350 mg).

**Table 3:** Baseline characteristics of patients

Patients included	27
Male, percentage (number of males/total number of patients)	78% (21/27)
Age in years, mean (standard deviation)	60 (13)
APACHE II score, mean (standard deviation)	23 (7.8)
Length of stay in intensive care unit in hours, median (interquartile range)	112 (59–158)
In-hospital mortality, percentage (number of deaths/total number of patients)	52% (14/27)

APACHE, Acute Physiology and Chronic Health Evaluation.

### Impedance and anthropometrics

Body composition measures are presented in Table 2. The ICC for TBF by impedance [7], TBF by the Durnin and Womersley [8] tables, TBF by Womersley [9] formula, TBF by James [10], and TBF by von Döbeln [11] and Deurenberg and colleagues [12] was 0.94 (95% confidence interval [CI] 0.89 to 0.97).

### Course of therapeutic hypothermia

The lengths of time needed to achieve temperature targets are shown in Table 4. Illustrative temperatures during the hypothermia process are shown in Table 5. The mean difference between the temperatures at admission and at the end of the active cooling period was 2.1°C. (95% CI 1.6 to 2.6). The change in temperature toward hypothermia, mean (SD) 2.1°C (1.1), was smaller compared with the change in temperature from hypothermia toward normal temperature, mean (SD) 2.9°C (0.8); mean difference 0.8°C (95% CI -1.3 to -0.3). None of the patients encountered critical events during the course of therapeutic hypothermia.

**Table 4:** Different periods during the cooling process

Time periods/duration (n = 27)	Minutes
Time from admission to start of cooling	41 (16–76)
Time from admission to target temperature	191 (105–382)
Duration of active cooling	1,525 (1,432–1,740)
Time from start of cooling to target temperature	152 (64–275)
Time from termination of active cooling to temperature of ≥ 36°C	840 (514–1,080)

All values are expressed as median (interquartile range).

**Table 5:** Temperature profile of cooled patients (n = 27)

	Degrees Celsius
Temperature at start of cooling, mean (SD)	35.3 (1.05)
Temperature drop per hour during active cooling, median (IQr)	0.5 (0.11–1.0)
Minimum temperature during active cooling, mean (SD)	31.8 (0.74)
Maximum temperature during active cooling, mean (SD)	34.4 (0.40)
Temperature at termination of active cooling, mean (SD)	33.1 (0.75)
Temperature rise per hour after termination of active cooling, median (IQr)	0.21 (0.16–0.33)
Total temperature rise until normal temperature (36°C), mean (SD)	2.9 (0.75)

IQr, interquartile range; SD, standard deviation.

### Time to achieve hypothermia

Hazard ratios, corrected for age and gender, for the time to target temperature were 0.98 (95% CI 0.95 to 1.0; *P *= 0.07), 0.93 (95% CI 0.85 to 1.0; *P *= 0.15), and 0.19 (95% CI 0.03 to 1.0; *P *= 0.06) for body weight, body mass index, and body surface area, respectively. Hazard ratios, also corrected for age and gender, for TBF by impedance, TBF by Durnin, and waist-to-hip ratio were 0.98 (95% CI 0.93 to 1.0; *P *= 0.42), 0.93 (95% CI 0.87 to 0.99; *P *= 0.03), and 0.004 (95% CI 0.0 to 6.63; *P *= 0.15), respectively. Cooling velocity was not affected by the use of rocuronium; the drop per hour during active cooling with the use of rocuronium (n = 20) was median 0.49°C per hour (95% CI 0.10 to 1.01) versus 0.39°C per hour (95% CI 0.36 to 0.96) without the use (n = 7) of rocuronium (*P *= 0.83).

## Discussion

Only one of the body composition parameters was associated with the time to achieve target temperature with hypothermia. TBF (anthropometric by Durnin's table) showed a significant but slight increase in the time needed to achieve target temperature if TBF increases. As none of the other highly concordant measurements showed a relationship with the achievement of the target temperature, the association might have no clinical relevance. From our data, we cannot justify the adjustment of cooling practice solely based on body composition. Considering the time needed to reach the target temperature, additional research into other factors is necessary.

According to Polderman [13], younger patients react earlier and with greater intensity and effectiveness to changes in body temperature than older patients. Polderman also implies that the surface cooling of obese patients will be more difficult and require significantly more time to achieve the target temperature. We could not confirm these hypotheses. Maybe because the ice-cold fluid infusion is administered by (estimated) body weight, the effect of the insulated properties of fat is less important than when cooling is done by surface cooling only. It seems reasonable, though, that the influence of body composition is higher when only external cooling techniques are used rather than internal cooling techniques. However, since a combination of internal and external methods was used in the present study, we are not able to answer this question.

The use of rocuronium reduces shivering and, therefore, cooling velocity may be increased with its use. There was, however, no difference in cooling velocity between patients who did receive rocuronium and those who did not. Also, patients not receiving rocuronium might have subclinical shivering, which can be detected only by electromyography, and therefore these patients have a reduced cooling velocity. Since we did not perform electromyography in our patients, we cannot confirm this hypothesis in our study.

Bernard and colleagues [14] succeeded in decreasing body temperature from 35.5°C to 33.8°C within 30 minutes. That is a temperature drop of 3.4°C per hour. Only one patient within our series met this criterion. In this particular study, patient characteristics such as weight and body composition were not mentioned. It is important to acknowledge that the main limitation of this study is the small sample size and subsequent lack of power. This is due mainly to the length of time it took to recruit patients into the study.

## Conclusion

The time to reach target temperature seems not to be influenced (or at most only partly) by body composition. There might be other factors like systemic vascular resistance, basic metabolism, shivering, or other factors of influence that cannot be measured within this study but should be subjected to further research.

## Key messages

• From our data, we cannot justify the adjustment of cooling practice solely based on body composition.

• Time to reach target temperature seems not to be influenced (or at most only partly) by body composition.

## Abbreviations

CI = confidence interval; ICC = intraclass correlation coefficient; ICU = intensive care unit; OHCA = out-of-hospital cardiac arrest; SD = standard deviation; TBF = total body fat.

## Competing interests

The authors declare that they have no competing interests.

## Authors' contributions

JJJ performed data acquisition and drafted the manuscript. FP participated in the study design and facilitated data acquisition. JMB participated in study design and conceptualization and carried out the statistical analysis. EMHM-V helped with the body composition concepts. MJS and MBV conceived of the study, participated in its design and coordination, and helped to draft the manuscript. All authors read and approved the final manuscript.
